# Transgenic approaches to altering carbon and nitrogen partitioning in whole plants: assessing the potential to improve crop yields and nutritional quality

**DOI:** 10.3389/fpls.2015.00275

**Published:** 2015-04-22

**Authors:** Umesh P. Yadav, Brian G. Ayre, Daniel R. Bush

**Affiliations:** ^1^Department of Biological Sciences, University of North Texas, Denton, TX, USA; ^2^Department of Biology, Colorado State University, Fort Collins, CO, USA

**Keywords:** assimilate partitioning, sugar transport in plants, amino acid transport, crop yield, nutritional value

## Abstract

The principal components of plant productivity and nutritional value, from the standpoint of modern agriculture, are the acquisition and partitioning of organic carbon (C) and nitrogen (N) compounds among the various organs of the plant. The flow of essential organic nutrients among the plant organ systems is mediated by its complex vascular system, and is driven by a series of transport steps including export from sites of primary assimilation, transport into and out of the phloem and xylem, and transport into the various import-dependent organs. Manipulating C and N partitioning to enhance yield of harvested organs is evident in the earliest crop domestication events and continues to be a goal for modern plant biology. Research on the biochemistry, molecular and cellular biology, and physiology of C and N partitioning has now matured to an extent that strategic manipulation of these transport systems through biotechnology are being attempted to improve movement from source to sink tissues in general, but also to target partitioning to specific organs. These nascent efforts are demonstrating the potential of applied biomass targeting but are also identifying interactions between essential nutrients that require further basic research. In this review, we summarize the key transport steps involved in C and N partitioning, and discuss various transgenic approaches for directly manipulating key C and N transporters involved. In addition, we propose several experiments that could enhance biomass accumulation in targeted organs while simultaneously testing current partitioning models.

## Introduction

The principal components of plant productivity and nutritional value, from the standpoint of modern agriculture, are the acquisition and partitioning of organic carbon (C) and nitrogen (N) compounds among the various organs of the plant. The initial step of C acquisition is the fixation of atmospheric C dioxide by photosynthesis followed by sugar partitioning to the non-photosynthetic tissues. N is primarily absorbed as inorganic salts from the soil solution and incorporated into amino acids in the root or shoot for subsequent transport to import-dependent tissues. These “sinks” are the primary consumers of newly assimilated sugars and amino acids, and include many essential organ systems such as expanding leaves, roots, seeds and fruits, storage tissue and organs, and secondary growth. As much as 80% of the C assimilated in mature leaves, and comparable amounts of amino acids, are exported in the phloem to satisfy the metabolic needs of the heterotrophic tissues (Kalttorres et al., 1987). Understanding the mechanisms and regulation of C and N partitioning to these heterotrophic tissues has always been a central theme to improving crop productivity. Moreover, human manipulation of C and N partitioning is evident from the earliest examples of crop domestication. A good example is the selective concentration of carbohydrate and storage proteins in the larger kernels and inflorescences of maize during domestication from ancestral teosinte as much as 10,000 years ago (Doebley, 2004). Likewise, the dramatic yield increases of the green revolution were, among other advances, based on the selection of smaller stature plants that invested more biomass in reproductive tissue versus vegetative growth. Indeed, yield trials of early twentieth century winter wheat versus late twentieth century cultivars grown in a common garden experiment showed that C assimilation per acre was the same for both varieties, yet the yields of the modern cultivars outperformed the early varieties by 40% because more C was directed into reproductive growth (seeds) versus vegetative tissues (Gifford et al., 1984). Yield enhancement of harvested organs continues to be a primary objective, whether the intent is edible organs for feed/food consumption, fiber for textiles and composites, wood for pulp, paper and building, or general biomass for biofuels. Nutritional and other quality enhancements of feed/food crops are also primary objectives that are in part dependent on C and N partitioning.

The last several decades have produced substantial biochemical and molecular advances in our understanding of cell-to-cell and long-distance partitioning of sugars and amino acids in plants. These advances have created novel opportunities to manipulate partitioning pathways to enhance yield and/or nutritional quality. The basic premise of these approaches is that altering the expression of the transporters for a desired sugar or amino acid will result in the preferential accumulation of that compound (or a derivative thereof) in a target organ or tissue. The success of these efforts are predicated on accurate knowledge of the physiological and biochemical processes involved to predict the outcome. The goal of this review is to briefly summarize current understanding of the functional contributions of sugar and amino acid transport systems to plant growth and then focus on the limited number of studies where ectopic expression has been attempted. In many of these, the desired outcome has been at least partially achieved, and it is apparent that identifying the limitation—that is, why the full desired effect was not obtained—is necessary to achieve greater gains. Thus, the application of existing knowledge for biotechnology gains has in many cases identified new avenues of biological discovery in the complex interaction of nutrient use and growth. In addition, this review ventures experimental approaches for improving and targeting biomass partitioning to specific organs.

## Carbohydrates: Transport Mechanisms and Efforts to Manipulate Partitioning by Engineering Transport

Sucrose (Suc) is the primary form of reduced C transported long-distance in the vascular system of plants, and consequently has garnered the most attention. Suc is produced in the cytoplasm, either directly from the products of photosynthesis or from storage reserves. Once produced, it may remain in the cytoplasm to participate in other aspects of cellular metabolism or it may be compartmentalized, principally in the vacuole; it may pass through the symplasm—the collective cytoplasm of the plant—via plasmodesmata (PD); or it may undergo efflux to the apoplasm—the collective cell-wall space of the plant—and from there it may be taken up into the cytoplasm of an adjacent cell. As a relatively large polar solute, Suc requires protein transporters for efficient movement across membranes. Where the prevailing concentration gradients mandate energized transport, Suc/H^+^ symporters (Suc uptake transporters, SUT; or Suc uptake carriers, SUC; the SUT designation is used here, unless referring to a specific gene) that utilize the proton motive force for Suc/H^+^ symport are well characterized biochemically, genetically, and physiologically (Bush, 1993; Braun and Slewinski, 2009; Ayre, 2011). Those SUTs that are best characterized function at the plasma membrane to pump Suc into cells from the apoplasm, but members of one SUT sub-family localizes to the tonoplast to use the prevailing proton gradient to move Suc from inside the vacuole out to the cytoplasm (Endler et al., 2006; Reinders et al., 2008; Eom et al., 2011). Suc/H^+^ antiport to load Suc into vacuoles is supported by physiological studies and patch clamping with mutant *Arabidopsis* lines indicate a role for TMT1 and TMT2 in this process (Schulz et al., 2011). Under conditions where passive transport would suffice, a new family of sugar transporters, SWEETs, were recently identified that have mechanisms characteristic of facilitated diffusion (Chen et al., 2010, 2012; Chen, 2014).

**FIGURE 1:**
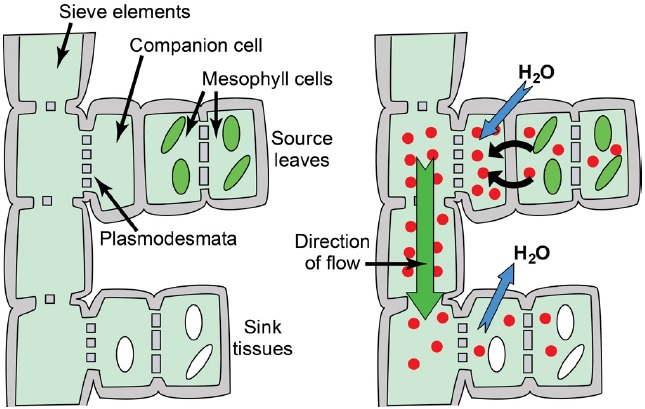
**Phloem anatomy and osmotically generated-pressure flow.** *Left*: Schematic of phloem connecting source leaves (represented as mesophyll cells with green chloroplasts) and heterotrophic sink tissues (represented as cells with white amyloplasts). Sieve elements are connected end to end to form sieve tubes, and companion cells are connected to sieve elements via plasmodesmata/pore units. The extent to which companion cells are connected to surrounding mesophyll/parenchyma cells varies substantially between loading strategies. *Right*: Hydrostatic pressure is established in source leaves by phloem loading—the accumulation of solute (red circles; primarily photoassimilate, but also amino acids, potassium, and others solutes) in the phloem—and the resulting osmosis of water. An exception is passive loading, in which solute and hydrostatic pressure is high throughout the source leaf (see Figure 4). Hydrostatic pressure drops in sink tissues as solute is used for growth and metabolism, and water dissipates. Bulk flow of water and nutrients through the sieve tubes follows the hydrostatic pressure gradient from source to sink.

**FIGURE 2:**
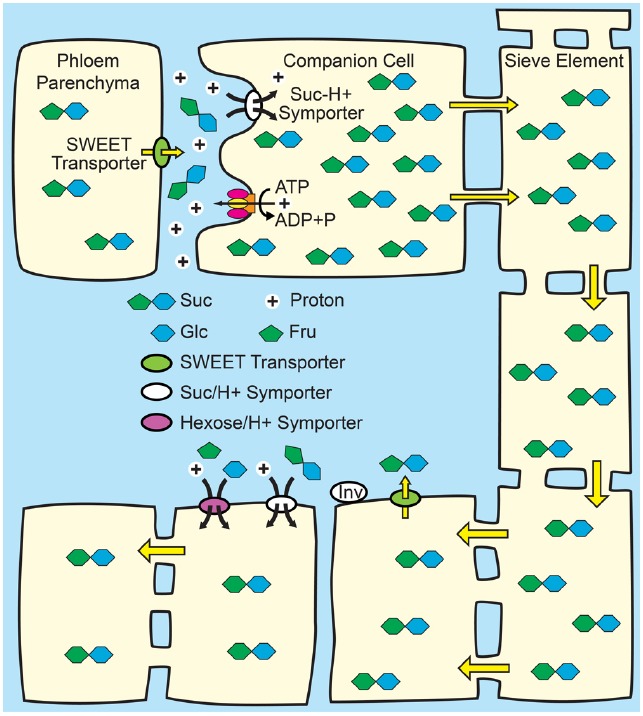
**Phloem loading from the apoplasm.** *Top*: Suc from mesophyll cells—principally phloem parenchyma cells—enters the apoplasm in the vicinity of the companion cell/sieve element complex via SWEET transporters. Suc, and in some species, polyols, is then loaded into the companion cell/sieve element complex up a thermodynamically unfavorable concentration gradient by Suc/H^+^ symporters (SUTs) energized by the proton motive force. The proton motive force is generated by the hydrolysis of ATP at the plasma membrane by proton pumping ATPases. Osmosis generates hydrostatic pressure that pushes the phloem sap toward sink tissues. *Bottom*: In sink tissues, solute is unloaded from the sieve elements. Post-phloem transport may be through the symplasm via plasmodesmata, or solute may efflux to the apoplasm for subsequent transmembrane transport in adjacent cells. Suc in the apoplasm of sink tissues may be taken up by SUTs, or maybe hydrolyzed to Glc and Fru by cell wall invertases (INV) and taken up by hexose/H^+^.

**FIGURE 3:**
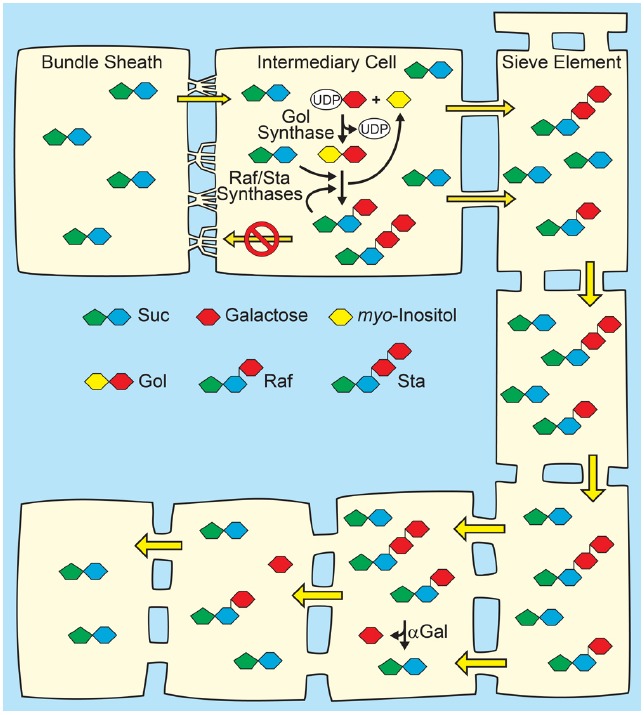
**Polymer-trap model for loading through the symplasm.** *Top*: Suc from mesophyll diffuses into intermediary cells (specialized companion cells) through highly branched plasmodesmata situated between the intermediary cells and bundle sheath cells, where a portion is converted to raffinose family oligosaccharides (RFO), raffinose (Raf) and stachyose (Sta), by the sequential action of Raf synthase and Sta synthase. The galactosyl donor for RFO synthesis is galactinol (Gol) created from UDP-galactose and myo-inositol by Gol synthase. The RFOs appear to be too large to diffuse back through the plasmodesmata into the mesophyll. Conversion of Suc to RFO favors the continued passive entry of Suc while RFO accumulation generates hydrostatic pressure by osmosis. *Bottom*: In sink organs, RFOs move through the post-phloem symplasm. Hydrolysis by alkaline α-galactosidases (αGal) and other enzymes convert RFOs to Suc and Glc, which can also partition via the apoplasm (not shown).

**FIGURE 4:**
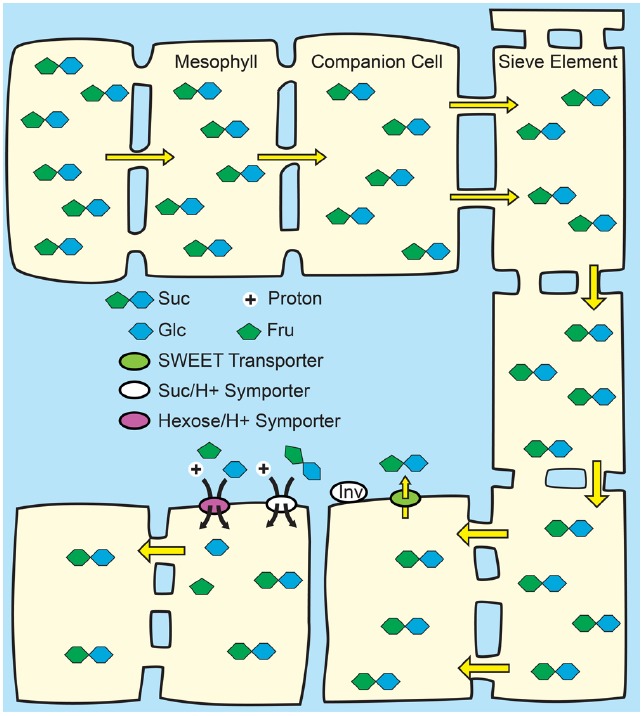
**Passive loading through the symplasm.** *Top*: Source leaves of species that use passive loading have regular plasmodesmata connections from the mesophyll cells through to the sieve elements and have high solute concentrations throughout the leaf. Consequently, solute is able to move relatively freely, and all cells have equally high hydrostatic pressures: there is not an energized concentrating step at the mesophyll/phloem interface. *Bottom*: Lower hydrostatic pressures in sink organs permit bulk flow through the sieve elements. Suc is unloaded from the sieve elements in sink organs through symplasmic or apoplasmic routes, as required by the specific tissues.

The primary fate of photoassimilated C is long-distance transport from photoautotrophic source leaves to heterotrophic organs. C partitioning via long-distance transport of sugars in the phloem has been extensively and recently reviewed (Turgeon and Wolf, 2009; Ayre, 2011; Braun et al., 2014; Chen, 2014) and is thus only surveyed here. Among mesophyll cells, Suc appears to move cell to cell relatively freely through the PD of the symplasm, and then enters the minor-vein phloem for long-distance transport through the sieve tube elements by bulk flow (Figure 1). Bulk flow occurs when a hydrostatic pressure difference between source and sink tissues is large enough to drive flux through the sieve elements, with the pressure difference primarily energized by solute accumulation in source-leaf phloem. Establishing a sufficient solute concentration in source phloem is generically referred to as phloem loading, and three mechanisms are proposed: (1) In apoplasmic phloem loading, Suc exits the mesophyll symplasm in the vicinity of the phloem via SWEET proteins located principally on the plasma membrane of presumptive phloem parenchyma cells. Suc is then accumulated against a concentration gradient from the apoplasm into the companion cell/sieve element complex of the phloem by SUTs (Figure 2). Because uptake is energized by the proton motive force, Suc can accumulate to high levels such that the total solute concentration in source phloem can readily exceed 1 Osm. Sugar alcohols (polyols) are prominent transport sugars in some species, and appear to be loaded into the phloem from the apoplasm by proton symporters in a mechanism equivalent to Suc loading from the apoplasm (Noiraud et al., 2001; Gao et al., 2003; Reidel et al., 2009). (2) In the polymer trap mechanism, Suc diffuses from the mesophyll symplasm into the phloem through specialized, highly-branched PD, and a portion is then converted to raffinose family oligosaccharides (RFO), primarily the trisaccharide raffinose and tetrasaccharide stachyose, but also, in some species, longer chain oligosaccharides verbascose and ajugose. These highly branched PD, located between bundle sheath and intermediary cells (specialized companion cells), are proposed to have a precise size exclusion limit that allows diffusion of Suc but not larger RFOs (Figure 3). RFO synthesis in the intermediary cells thus maintains a diffusion-friendly Suc concentration while generating the high osmolarities necessary for the hydrostatic pressure that drives long-distance transport through the sieve tubes (Turgeon, 1996). (3) In passive loading, source leaf mesophyll cells accumulate high concentrations of Suc, which enters the phloem passively through regular PD (Figure 4). In this mechanism, there is not an energized step for concentrating solute into the companion cell/sieve element complex, and the high turgor required for bulk flow through the sieve elements is maintained throughout the leaf (Rennie and Turgeon, 2009; Slewinski and Braun, 2010). It should be noted that some species can employ multiple phloem loading pathways in the same vascular bundle (Voitsekhovskaja et al., 2009) and that different mechanisms can be favored depending on the plant’s specific needs (Gil et al., 2011).

Phloem unloading occurs all along the transport path to nourish lateral tissues and storage reserves, and occurs extensively in the sink organs. Unloading may be by efflux across plasma membranes to the apoplasm, or through the symplasm via PD, or both routes may be used in combination. Within the recipient tissues, symplasmic continuity with the CC/SE complex can be extensive for efficient post-phloem partitioning via PD, or isolated domains may force apoplasmic transport (Patrick, 1997). Generalizing, growing vegetative tissues, such as young leaves and root tips, tend to have extensive symplasmic unloading (Oparka et al., 1999; Stadler et al., 2005b). This contrasts with developing seeds, where symplasmic isolation between maternal and filial tissues forces apoplasmic unloading: short distance symplasmic unloading occurs from the phloem strands entering the integument/testa tissue, from where nutrients enter the apoplasm for subsequent uptake into the embryo and endosperm (Wang and Fisher, 1994; Patrick and Offler, 2001; Stadler et al., 2005a). In addition, the principal route of unloading, and the extent of the symplasmic and apoplasmic domains in the post-phloem pathway, may change during organ development, as exemplified in maturing fruit and developing embryos (Ruan and Patrick, 1995; Ruan et al., 2001; Kim et al., 2005). Once released to the apoplasm, Suc may be retrieved back into the phloem or into adjacent cells by SUTs, or may be hydrolyzed by cell wall invertases, and recovered by hexose/H^+^ transporters.

The complexity of the loading and unloading pathways involved in C partitioning, and their importance to plant physiology, are in part reflected in the size of the gene families involved. *Arabidopsis* has nine SUTs arranged in three sub-families, and monocots have an additional one or two families not represented in dicots (Sauer, 2007; Braun and Slewinski, 2009; Reinders et al., 2012). The monosaccharide transporter-like (*MST*) gene family of *Arabidopsis* has 53 members, arranged in seven sub-families. The best characterized of these sub-families, and the one with members that localize to the plasma membrane and are thus most likely involved in intercellular transport, is the sugar transporter (STP) family, which contains 14 members (Büttner, 2007, 2010). The SWEET family of mono- and disaccharide transporters contains 17 genes in *Arabidopsis* and 21 in rice, arranged in four clades, with clade I mediating mainly Glc import and export, and clade III transporting mainly Suc (Chen et al., 2010, 2012). Plants have three types of invertases, localizing to the cell wall/apoplasm, cytoplasm, and vacuole, respectively, with six members in the cell wall invertase subfamily of *Arabidopsis* (Roitsch and Gonzalez, 2004). This list only includes the major candidate participants in membrane-mediated C partitioning, and not those involved in polymer synthesis or PD function.

### Engineering Partitioning by Manipulating Apoplasmic Loading

Strategic manipulation of C partitioning may lead to more productive plants and enable specific organs to be targeted for enhanced yield. Ectopic expression of *SUTs* from constitutive or tissue-specific promoters, in either sink or source organs, has been attempted in various plants. These studies demonstrate a clear potential, but also emphasize that the natural patterns of Suc flux need to be considered. In potato, constitutive over-expression of spinach *SoSUT1* from the CaMV 35S promoter was used in an effort to increase transport to tubers (Leggewie et al., 2003). This gave higher levels of starch and lower levels of sugar in leaves, but had little impact on tubers yields. These effects were likely caused by futile cycling, in which Suc released to the apoplasm was recovered by mesophyll cells because the promoter drove expression in the mesophyll as well as in phloem. Ectopic expression of potato *StSUT1* in excised pea cotyledon storage parenchyma enhanced Suc influx in a *StSUT1*-dependent fashion (roughly twofold). However, Suc uptake into intact cotyledons was only ∼23%, and when biomass accumulation was measured, a similar difference between excised and intact cotyledons was observed (Rosche et al., 2002). This study showed that ectopic *SUT* expression can be used for targeted yield enhancements, but the authors also argue that since most Suc is absorbed at the cotyledon surface by epidermal transfer cells, the full effect of overexpressing *StSUT1* in storage parenchyma was not realized in intact seedlings since the “extra” transporters were not on the transfer cells exposed to apoplasmic Suc (Rosche et al., 2002). As another example of manipulating SUT activity for biotechnology, overexpression of a barley SUT (*HvSUT1*) using an endosperm-specific *Hordein B1* promoter in wheat grains increased levels of storage protein, showing that enhanced Suc transport has positive impacts beyond carbohydrate alone (Weichert et al., 2010). Finally, over-expression of rice *OsSUT5Z* in potato using a tuber-specific, class-I patatin promoter was reported to enhance tuber yield by increasing tuber numbers rather than tuber size. This was not the anticipated outcome, and the authors suggest that altering Suc flux stimulated development of more stolons (Sun et al., 2011).

The above studies emphasize constitutive or sink-specific manipulation, and attempt to “pull” more Suc into the target tissue. Enhancing phloem transport by manipulating *SUTs* involved in phloem loading has been put forward as a “push” mechanism to improve plant productivity. For example, increasing source to sink transport was proposed as a means to enhance crop productivity since more carbohydrate would be sent to sink organs for growth and/or storage, and there would be less Suc-mediated product inhibition on photosynthesis (Ainsworth and Bush, 2011). In addition, Suc loading is modulated, both up and down, in response to the physiological and environmental needs (Vaughn et al., 2002), and it was proposed that heterologous promoters that are uncoupled from the natural regulation may be useful to keep loading rates constantly high (Srivastava et al., 2009).

Despite this, there are few published attempts to enhance phloem transport by over-expressing SUTs in the phloem. The cDNA of *Arabidopsis AtSUC1* (Wippel and Sauer, 2012) and barley *HvSUT1* (Reinders et al., 2012) were cloned downstream of the companion cell-specific *AtSUC2* promoter, and both restored phloem loading in *Arabidopsis Atsuc2* –/– mutants. These studies showed foreign transporters could restore WT growth to the mutant, but detailed analysis of C transport and impact on growth was not pursued. In a different approach, *AtSUC2* cDNA was expressed from a companion cell-specific promoter derived from Commelina yellow mottle virus (*CoYMVp*) in a homozygous *Atsuc2-4* background (Srivastava et al., 2009). *CoYMVp* is as strong as the natural *AtSUC2* promoter, but is subject to different regulatory cascades: while SUT promoters naturally involved in phloem loading are repressed by Suc accumulation (Vaughn et al., 2002; Usadel et al., 2008), *CoYMVp* is activated by Suc (Dasgupta et al., 2014). This over-expression construct rescued *Atsuc2-4* mutants, showing that foreign promoters can maintain high levels of SUT activity in conditions where expression might normally be repressed (Srivastava et al., 2009).

A more recent study fused *CoYMVp* to alternative *SUTs*, with the aim of keeping phloem loading levels high by uncoupling the natural transcriptional control via the foreign promoter and potential post-translational control with different proteins. Of the *SUTs* tested, *AtSUC1*, *AtSUC2*, and *ZmSUT1* (a *Zea mays* gene from a different *SUT* subfamily) rescued phloem loading in the *Atsuc2-4* mutant; several other *SUT* genes implicated in high affinity Suc uptake were unable to restore efficient phloem transport (Dasgupta et al., 2014). When *SUTs* that did rescue *Atsuc2-4* were overexpressed in WT plants, enhanced phloem loading and transport to heterotrophic organs was evident, but improved growth and primary productivity was not observed. Rather, the plants were stunted. Separate research suggested a link between sugar transport and phosphate requirements (Lei et al., 2011), and this was tested in lines overexpressing *SUTs* from *CoYMVp*. The growth inhibition was accompanied by increased expression of phosphate-starvation induced genes, and was reversed by providing a higher supply of external phosphate (Dasgupta et al., 2014). These findings argue that “hyperloading” the phloem by overexpressing *SUTs* have the detrimental effect of disrupting C/phosphate homeostasis. The implications for agriculture are that efforts to enhance photosynthesis and growth by increasing phloem transport may be imperiled by the plant’s perception that it needs more P, unless the links between C and P homeostasis are better understood and uncoupled.

What future experiments could be done to enhance partitioning via apoplasmic loading? Molecular characterization of SWEET and SUT transporters provides, in principle, a complete framework to test hypotheses on the strategic release of Suc from one cell and loading into the adjacent cell. An obvious experiment is to overexpress *SWEETs* in phloem parenchyma cells, particularly *Arabidopsis SWEET11 and SWEET12* (Chen et al., 2012), for enhanced Suc efflux to the apoplasm with simultaneous *SUT* overexpression in companion cells for increased uptake, and then test the impact on growth and whole plant C partitioning. Precise cell-specific deployment will be critical for accurate interpretation of the results, and a caveat to the success of these experiments is the resolution with which the outcome is measured (see below). Simply expecting larger plants in general, or larger target organs, is naïve, even though this may be the desired outcome from an applied standpoint. Instead, these experiments will more likely help identify rate-limiting steps in the overall process of whole-plant partitioning, and thus lead to new prospects (targets) for manipulation, and uncover unrecognized interactions of C homeostasis and other aspects of physiology. A corollary to these experiments aiming to enhance productivity are those that disrupt partitioning to test prevailing and alternative models of C allocation.

Manipulation of source-leaf phloem loading would energize the entire pathway, but by itself does not target resources to desired (i.e., harvested) organs. Targeted sink-specific manipulations may therefore be more fruitful for enhancing yield of specific organs, as evident from overexpression of *StSUT1* in storage parenchyma of pea cotyledons and *HvSUT1* in wheat endosperm, described above. But here too, the complete pathway of Suc unloading, from the sieve elements to the final recipient cells need to be considered (Patrick, 1997). For example, developing embryos and endosperm are symplasmically isolated from maternal tissues, and all nutrient transfer has two obligate membrane transport steps: efflux from maternal cells and influx into filial cells (Zhou et al., 2009). Tissue-specific *SWEET* over expression in integuments/testa of developing seeds may enhance sugar availability at the interface between maternal and filial tissues, but optimally positioned uptake carriers may be required to take advantage of the available resources.

Similarly, Suc unloading to the apoplasm in sink organs is associated with hydrolysis by cell wall invertase and uptake of the resulting hexose by hexose transporters. This is particularly prevalent in fruits undergoing rapid expansion and ripening (Ruan and Patrick, 1995; Patrick, 1997; Zhang et al., 2006, 2007; Jin et al., 2009). As the fruit ripens and accumulates more solutes (simple sugars, etc.), apoplasmic unloading prevents symplasmic “backflow.” In addition, hydrolysis of Suc to Glc and Fru doubles the osmolarity to benefit hydrostatic expansion; enhances the Suc gradient between symplasm and apoplasm to favor further passive unloading; and also prevents the retrieval of unloaded Suc back into the phloem by SUTs, which are expressed all along the phloem path.

Although the potential for manipulating the “pull” capacity of sinks is clear, little is known on how manipulation of hexose transporters could be advantageous to increase plant yield. Over-expression of sugar transporter *AtSTP13* in *Arabidopsis* seedlings led to increases in Glc uptake, and also led to increases in the sugars and biomass throughout the plants (Schofield et al., 2009). In tomato, RNAi-mediated knockdown of the high affinity hexose transporter gene *LeHT* led to a 55% decrease in fruit hexose accumulation, implicating *LeHT* in driving accumulation of hexoses into storage parenchyma cells during tomato fruit ripening (McCurdy et al., 2010). Among those hexose transporters that have been characterized genetically, many of the knock out mutants have no visible phenotype. The expression patterns of *Arabidopsis STP* genes, as determined by classical hybridization and PCR approaches, as well as analysis of genome-wide transcriptome data, overwhelmingly place *STP* expression in various sink organs (Büttner, 2010) and this speaks strongly to their potential for targeting biomass to specific sinks by transgenic approaches. Genetic manipulation of additional sugar transporter genes using constitutive and tissue-specific promoters is needed to test this potential. Since manipulating one aspect of the transport system may have limited impact, coupling the targeted expression of genes encoding SWEETs, invertases, and hexose transporters may be more fruitful to capture the full potential of this strategy.

However, manipulating the transporters involved in apoplasmic phloem loading and unloading carries the inherent risk of increasing the apoplasmic concentrations of Suc and other nutrients used by pathogens. Pathogens have been shown to enhance SWEET expression to make reduced C more available (Chen et al., 2010, 2012; Chen, 2014). Several lines of genetic and metabolic evidence implicate sugar transporters in plant defense responses during plant-pathogen interactions. This includes reprogramming carbohydrate metabolism at the site of infection for reduced Suc export, increased Suc hydrolysis by cell wall invertase, and enhanced import of hexose by hexose transporters (Proels and Hückelhoven, 2014).

### Engineering Polymer Trapping Metabolism

Other efforts to enhance phloem loading and long-distant transport have relied on the fact that there are different phloem loading mechanisms, and have attempted to superimpose an alternative mechanism on top of the natural mechanism of the host plant. Specifically, polymer trapping biochemistry was introduced to two species, potato (Hannah et al., 2006) and *Arabidopsis* (Cao et al., 2013), both of which load from the apoplasm with SUTs. The aims of these experiments were twofold: (1) to assess the efficiency of RFO synthesis in the phloem and (2) to gage the efficiency with which RFO from the companion cells enters the translocation stream for long-distance transport. In addition, growth and development of the engineered plants were monitored. In both studies metabolic engineering was used to produce galactinol and raffinose in companion cells. In *Arabidopsis*, stachyose was also engineered. Both studies used companion cell-specific promoters to express genes encoding galactinol synthase and raffinose synthase, and in *Arabidopsis*, stachyose synthase. Thus, all the RFO biosynthesis occurred in the phloem after the SUT-mediated phloem loading step. Despite the high concentrations of Suc available in these cells, RFO synthesis and transport in both studies was low. When *Arabidopsis* was photosynthetically labeled with ^14^CO_2_ for 20 min followed by a 10 min chase period, only 2% of the label was incorporated into RFO, while Suc contained up to 80% and up to 30% was in Glc and Fru. This outcome is surprising and in sharp contrast to plants that transport RFO naturally, such as coleus (Turgeon and Gowan, 1992), cucurbits (Beebe and Turgeon, 1992), and catalpa (Otto, 1968; Turgeon and Medville, 2004), in which RFOs become quickly labeled to high specific activity.

Based on current models, this result is puzzling, and neither study had a satisfying explanation for the low rates of RFO synthesis other than to suggest that RFO biochemistry, especially in relation to phloem transport, was not straightforward. RFO synthesis should be efficient since apoplasmic loaders have ample reduced C in the companion-cell cytoplasm and the engineered proteins are thought to localize to the cytoplasm (Keller and Pharr, 1996); transport should be efficient because the PD-pore units between companion cells and sieve elements are open to diffusion of 10 kDa dextrans (Kempers and Van Bel, 1997; Knoblauch and Van Bel, 1998) and 67 kDa proteins (Stadler et al., 2005b). Further work on the biochemistry in companion cells of apoplasmic loaders and intermediary cells of polymer trap loaders is required to resolve why apoplasmic loaders do not effectively produce and transport RFOs. The precursors for galactinol, UDP-Gal and myo-inositol, or flux through the pathways leading to these, may be insufficient for higher level production.

Alternatively, the inability to produce high levels of RFO in companion cells may be a cell biology problem rather than a biochemical problem. In addition to many highly branched PD, intermediary cells of plants employing the polymer trap mechanism have many small vacuoles and extensive endomembrane systems, and are larger than companion cells found in similarly-sized leaf veins of plants that load from the apoplasm (Turgeon et al., 1993, 2001). A potential function for the extensive internal membranes and vacuoles in intermediary cells has not been put forward, but low rates of RFO synthesis in companion cells suggests a role for these membranes in RFO synthesis. Enzyme localization, stability, and/or interaction with cellular co-factors in companion cells relative to intermediary cells could therefore be a problem. It is worth noting that true intermediary cells and polymer trapping appear to have evolved independently multiple times, arguing that the internal structures are essential for function, and not species-specific characteristics (Turgeon et al., 2001).

**FIGURE 5:**
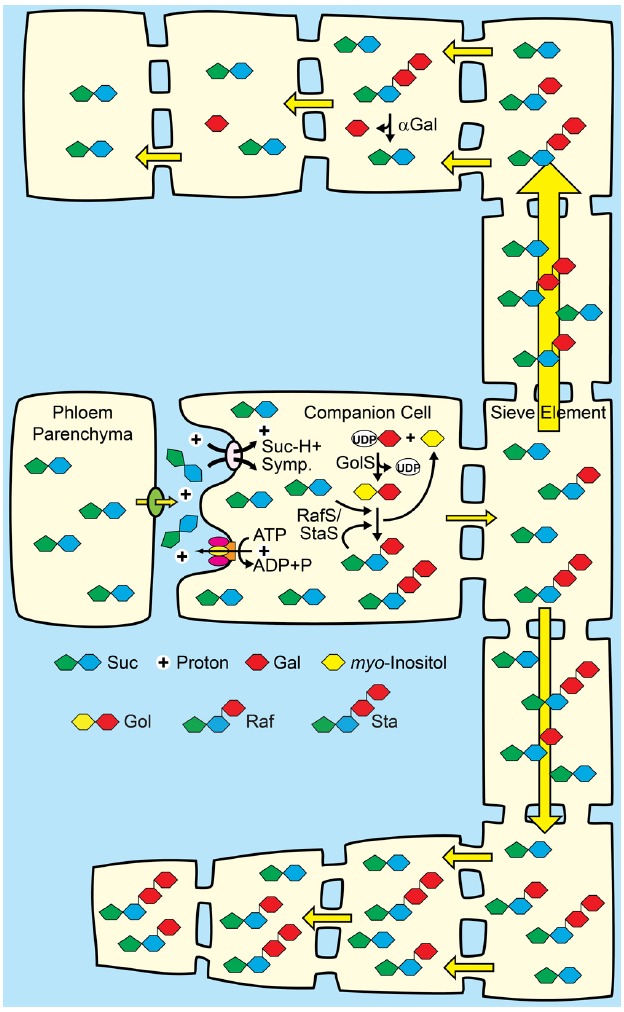
**A proposal for combining apoplasmic loading with polymer trap biochemistry and sink-specific digestion of RFO sugars.** *Middle*: Companion cells of source leaves loading from the apoplasm with Suc/H^+^ symporters are engineered to convert a portion of Suc to raffinose family oligosaccharides (RFO). See Figures 2 and 3 for details. *Top*: Target sinks engineered to efficiently catabolize RFOs using tissue-specific expression of genes encoding alkaline α-galactosidases (αGal) and galactose metabolizing enzymes. The hydrostatic pressure drops as the engineered sugars are catabolized. *Bottom*: Off-target sinks that are not engineered to catabolize RFOs accumulate the RFO and have a relatively high hydrostatic pressure. Direction of transport is based on hydrostatic pressure gradients, and engineered sinks are predicted to receive more sap (symbolized by relative arrow size). Engineered sinks are also predicted to compete better for resources and show better growth (symbolized by relative cell size).

If RFO synthesis in companion cells could be engineered to levels that contribute to hydrostatic pressure in the source leaf, then efficient RFO metabolism/utilization could be engineered in specific sinks for targeted partitioning (Figure 5). Expression of an alkaline α-galactosidase gene specifically in desired sink organs (Carmi et al., 2003), for example, would convert transported RFOs to Suc and galactose, which could then be converted to Glc (Barber et al., 2006; Dai et al., 2006) to enter “regular” pathways of primary metabolism. Since RFOs are not abundant transport sugars in apoplasmic loading species, competing, off-target sinks not engineered for efficient RFO catabolism may accumulate the RFO sugars (Figure 5). The principle here is to alter the hydrostatic pressure gradients that drive phloem transport throughout the plant: RFO utilization in target organs will result in low hydrostatic pressures and efficient nutrient import, whereas RFO accumulation in competing sinks that cannot metabolize the RFOs will have higher hydrostatic pressure that will decrease bulk flow to that tissue. With the strength of competing sinks reduced, more nutrients may be available for biomass accumulation in target sinks.

As a corollary to superimposing polymer trap chemistry on apolasmic loaders, superimposing apoplasmic loading onto intermediary cells may enhance partitioning in species that load by the polymer trap mechanism. Species that phloem load by polymer trapping do not have an obligate need for SUTs or Suc in the apoplasm (McCaskill and Turgeon, 2007). Notwithstanding, Suc is present in the apoplasm and many species with intermediary cells also have “regular” companion cells that express genes encoding SUTs (McCaskill and Turgeon, 2007; Voitsekhovskaja et al., 2009). Cucurbits, and probably other polymer trap species, appear to be able to switch between apoplasmic and polymer trap loading under certain conditions, particularly virus infection (Gil et al., 2011). Engineering *SWEET* expression in phloem parenchyma and *SUT* expression in the intermediary cells may increase Suc availability for RFO synthesis and consequently improve phloem loading and transport. It would also test predictions of the polymer trap model. Polymer trapping proposes that the branched PD allow Suc to diffuse down its concentration gradient. If this is correct, it follows that Suc brought into intermediary cells by SUT mediated loading should be able to diffuse back out. If on the other hand, Suc accumulates in the intermediary cells, it would speak against the polymer trap model.

### Engineering Passive Loading

Passive loading species are proposed to have the highest solute concentration in the mesophyll cells, and have PD with relatively large size-exclusion limits from the mesophyll all the way to the sieve tubes. Sugars and other nutrients are thus free to move along concentration gradients from the mesophyll into the sieve elements (Turgeon and Medville, 1998; Rennie and Turgeon, 2009). There is no energized concentrating step, and thus, speaking literally, there is no actual “loading” step. If this prevailing model is correct, specifically that PD with large size-exclusion limits connect the mesophyll and phloem cells to facilitate Suc movement without an apolasmic step nor a polymer trapping step, then it predicts that neither engineering a SWEET/SUT pairing to promote apoplasmic transport, nor polymer engineering to trap sugars in the phloem would improve loading and transport: The model predicts that any efforts to accumulate solute in the phloem would be thwarted by escape through the PD back to the mesophyll. Ironically, testing the passive-loading premise is exactly why these experiments should be conducted: If solutes do accumulate in the phloem after these manipulations, it would argue that the model is incorrect.

## Nitrogen: Transport Mechanisms and Efforts to Manipulate Partitioning by Engineering Transport

Although nitrate and ammonia are the primary forms of N acquired from the soil solution by plants, “amino acids are the currency of N exchange” in the physiology of plant growth (Bush, 1999). Inorganic N taken up from the soil is either assimilated into amino acids in the root, or they are transported to photosynthetically active leaf tissue in the xylem via the transpiration stream. Amino acids synthesized from inorganic N in the root are used for basic metabolic needs of root cells, but a substantial amount of those amino acids are transported into the xylem where they make their way to the photosynthetically active leaves in the transpiration stream. In the leaf, amino acids are used for basic metabolism and protein synthesis. However, as generally mature organs, these leaves do not need a large percentage of those amino acids for metabolism. Thus, amino acids arriving from the roots are transported into the leaf phloem where they move to the many heterotrophic tissues of the plant, that include young leaves just building the photosynthetic machinery, roots, stem tissues, storage organs, and developing seeds. When inorganic N from the soil moves directly to the leaves in the xylem, it is assimilated into amino acids in the mesophyll and then, much like the amino acids arriving from the root, they are transported into the phloem for systemic distribution to import-dependent sinks. Taken together, it is clear that amino acids are the primary form of transported N supporting the growth and development of multicellular plants (Bush, 1999).

The systemic distribution of amino acids requires the combined activity of many amino acid transport proteins. Example transport steps include export from xylem parenchyma in the root into the xylem transpiration stream, transport into mesophyll cells, export from mesophyll cells, transport into and out of the phloem, and transport into the heterotrophic cells of all the import-dependent sinks. Remarkable advances in our understanding of the transport properties and identity of plant amino acid transporters in the past 25 years was initially enabled by detailed biochemical descriptions using purified plasma membrane vesicles (Bush, 1993) followed by the successful cloning of a wide array of amino acid transporter gene families (Fischer et al., 1998; Ortiz-Lopez et al., 2000; Tegeder, 2012). To date, at least six families of amino acid transporters have been identified in plants with more than 60 genes encoding putative amino acid transporters. These transporters are functionally differentiated by their transport properties (such as substrate specificity and transport mechanism) and expression patterns that are regulated by both developmental and environmental cues (Liu and Bush, 2006; Tegeder, 2012, 2014). The majority of the transporters described to date are involved with active uptake into the cell, and only two reports describe bidirectional transporters (BAT1 and SIAR1) that may be involved in amino acid export (Dundar and Bush, 2009; Ladwig et al., 2012). BAT1’s putative role in amino acid export from the cell is based on its activity as a facilitated carrier and on a vascular tissue-localized expression pattern of a GUS expressing gene-trap inserted in the *BAT1* gene (Dundar and Bush, 2009). However, subsequent localization of the BAT1 protein using a BAT1::GFP reporter localized the carrier to the mitochondria, thereby suggesting a primary role in intracellular amino acid metabolism (Michaeli et al., 2011). In contrast, SIAR1 was localized to the plasma membrane of vascular tissue, thus supporting its role in amino acid export (Ladwig et al., 2012).

Given the complexity of amino acid circulation in the plant, a reasonable hypothesis suggests that altering the pattern and/or timing of the expression of one or more transporters could have a positive impact on plant growth and nutritional quality. For example, Rolletschek et al. (2005) documented a 20% increase in seed N content and seed size as a result of the ectopic expression of the *Vicia faba VfAAP1* amino acid transporter gene in the developing seeds of pea and *Vicia narbonensis* using the seed specific *legumin B4* promoter. These results suggest that amino acid transport into the storage parenchyma of the developing seed is rate limiting. Interestingly, there was also an overall increase in plant biomass, suggesting targeted changes in N allocation in one tissue can have a broader impact on plant growth. In a subsequent report, the transgenic peas were grown in field trials over two seasons (Weigelt et al., 2008). Seed N content was increased as before, but there were compensatory decreases in seed starch content and seed size. Transcript and metabolite profiling indicated the transgenic seeds were experiencing C limitations as more amino acids were being synthesized. Several pathways associated with coordinated C and N metabolism localized to the mitochondria were up-regulated, suggesting metabolic adjustments in response to a changing N:C ratio (Weigelt et al., 2008).

Tegeder and colleagues took a different approach in attempting to increase the sulfur content of legume seeds (Tan et al., 2010). In these experiments, the yeast *S*-methylmethionine permease 1 gene (*MMP1*) was expressed in transgenic pea under the control of the *Arabidopsis AAP1* promoter which directs expression throughout the phloem and in the seeds. *S*-methylmethionine (SMM) transport was targeted because it is identified as a major form of phloem mobile S in plants (Bourgis et al., 1999). *MMP1* expressing plants averaged a 33% increase in biomass, a 19% increase in seed number, a 31% increase in total seed N and a 19% increase in total seed S. There was also an average 67% increase in xylem and 60% increase in leaf SMM. Individual seed S content was unaltered. Analysis of the expression levels of genes involved in SMM biosynthesis suggested increased SMM synthesis in the root. That suggests SMM is transported to the leaf in the xylem, which was supported by the observed increased SMM xylem content. The increase in seed N content was driven by a 33% increase in phloem amino acid content. The surprising result in this experiment is the impact of altered SMM transport on N metabolism and yield. While S content per seed was unaltered, seed N content was increased, suggesting SMM abundance may have a regulatory impact on plant N metabolism. Indeed, SMM represents only 0.2% of xylem and phloem amino acids, suggesting it could be playing a role as a metabolic signal reporting on the S:N ratio in the plant. It is particularly interesting that two experiments that manipulate amino acid distribution in the plant (Weigelt et al., 2008; Tan et al., 2010) have had significant impacts on metabolic pathways that are associated with balancing the relative abundance of three important essential elements (N, C, and S). These observations are perhaps not surprising given the complex interactions between the functional roles of these central nutrients in plant metabolism.

Other approaches for manipulating N distribution in the plant might include ectopic expression of a desired amino acid transporter in the leaf phloem using a promoter that drives companion cell/sieve element specific expression. Under the expectation that anything loaded in the phloem moves with mass flow to actively growing sinks, this could increase the N content of harvested organs, such as mature seeds. Ectopic expression in the leaf phloem might be coupled to increased expression of a bidirectional transporter in the mesophyll that could increase the efflux of the desired amino acid into the apoplast. Likewise, one could simultaneously use a transgenic approach to increase the biosynthesis of a desired amino acid in the mesophyll, thereby increasing the pool size available for export. These approaches focus on the “push” side of the allocation pathway by attempting to increase the flux of an amino acid(s) to the sink tissue of the plant. Alternatively, one can increase the “pull” side of allocation by enhancing the uptake capacity of a desired sink, as Rolletschek et al. (2005) did with the ectopic expression of the *VfAAP1* gene.

**FIGURE 6:**
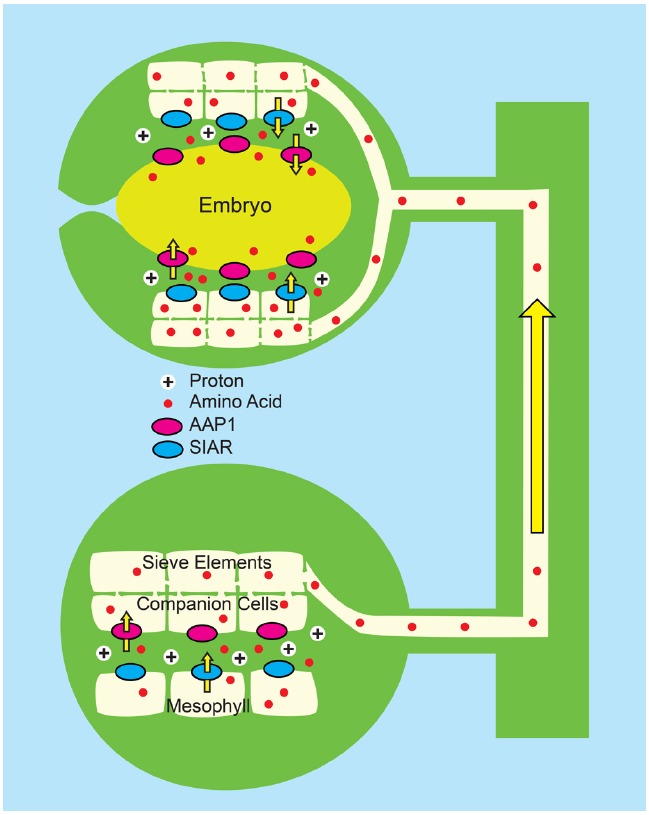
**Combined “push” and “pull” strategy for enhancing N transport to developing pea seedling (Zhang et al., 2014).** Pea plants (*Pisum sativum*) were engineered to overexpress *PsAAP1* (encoding Amino Acid Permease 1, magenta ovals) using the *Arabidopsis AtAAP1* promoter. In pea, the *AtAAP1* promoter promotes expression in the companion cell/sieve element complex of leaves, and in epidermal transfer cells of seed cotyledons. *Bottom*: In source leaves, amino acids (red circles) enter the apoplasm, possibly via the bidirectional transporter encoded by *SIAR1* (blue ovals). Uptake into the companion cell/sieve element complex is by endogenous PsAAP1, and is enhanced by PsAAP1 encoded from the transgene. *Top*: In the testa of developing seeds, amino acids unload from the sieve elements and the post-phloem symplasm most likely via *SIAR1* encoded transporters. Uptake into epidermal transfer cells of the cotyledons is by endogenous PsAAP1, and is enhanced by PsAAP1 encoded from the transgene.

In a combined strategy, the Tegeder lab (Zhang et al., 2014) has applied both a push and pull approach by ectopically expressing the pea *PsAAP1* gene in transgenic pea using the *Arabidopsis AtAAP1* promoter. In pea, the *Arabidopsis* promoter drove expression in the leaf companion cell/sieve element complex as well as the epidermal cells of the seed cotyledons, thereby generating enhanced amino acid transport capacity in both the source and sink tissues (Figure 6). The transgenic peas had an average 24% increase in biomass, compared to controls, a 228% increase in leaf phloem amino acids, a 35% increase in seed yield and a 6% increase in seed N. As previously observed, the activity of the transgenes had a pleotropic effect on overall plant N metabolism with greater N acquisition and significant increases in both leaf and root N content.

Transgenic manipulation of amino acid transport in plants has a real potential to increase the nutritional value of harvested tissue by increasing the overall N content and/or by targeted increases of specific amino acids, such as cysteine and methionine. However, it is clear from early transgenic experiments that altering amino acid distribution in the plant has a major impact on both N and C metabolism, which will require additional research to better understand the complex interactions between these macronutrients.

## Concluding Remarks

From the few published reports on efforts to manipulate C and N partitioning, it is evident that there is potential for agricultural improvements but also unexpected pleiotropic effects which limit the desired gains, and in some cases, have unpredicted effects on plant vigor. Of particular interest from both an applied and a basic science perspective are those reporting an influence on another nutrient, such as *HvSUT1* overexpression in endosperm increasing storage protein content (and consequently more N; Weichert et al., 2010); and conversely, *VfAAP1* expression in seeds increasing seed biomass (and consequently more C; Weigelt et al., 2008); *SUT* overexpression in companion cells impacting P requirements (Dasgupta et al., 2014); and MMP1-mediated transport of SMM in phloem enhancing seed N content (Tan et al., 2010). These studies point to the complex cross talk between nutrients in specific organs and throughout the entire plant. Also in relation to nutrient cross talk, it would be very interesting to combine manipulated C, N and other nutrient transport systems to look for synergistic effects.

Studying the phloem, phloem transport, and partitioning of nutrients directly is notoriously difficult (Turgeon and Wolf, 2009), and this limitation hampers interpretation of overexpression studies. Analysis of gross morphology (organ size, etc.) is an indirect metric. However, labeling experiments to monitor nutrient transport pathways, either directly with radiolabeled or stable isotopes, or indirectly with dyes or fluorescent proteins, have been, and continue to be, informative and essential. Key technological breakthroughs would be those that allow high-resolution analysis of C and N metabolite concentrations in intact tissues, preferably in real time. Nuclear magnetic resonance imaging has been applied to whole plants, mostly in the context of water relations, but has limited resolution and utility for low-concentration metabolites (Van As et al., 2009). Fluorescence-activated cell sorting and laser capture microdissection allow cell-specific analysis of transcripts and proteins, but have limited utility for small metabolites because of their high solubility and rapid turnover. Imaging mass spectrometry using matrix-assisted laser desorption/ionization (MALDI-IMS) is an emerging technology for spatial metabolomics in tissue sections (Zaima et al., 2010; Horn et al., 2012; Horn and Chapman, 2014). MALDI-IMS resolution is currently at the multiple-cell level and could, in principle, resolve differences across loading/unloading zones—for example, between integuments and cotyledons of the embryo—but the tissue needs to be suitably fixed to overcome the problems of diffusion and metabolism. FRET (Förster resonance energy transfer) nanosensors are engineered proteins that change their fluorescence emission wavelength when a specific metabolite is bound, and have been used to monitor changes in Glc and Suc levels in living plant tissue in real time (Deuschle et al., 2006; Chaudhuri et al., 2008; Chen et al., 2012). Nanosensors also exist for various amino acids (Okumoto et al., 2005; Gruenwald et al., 2012; Zhang et al., 2013). Their continued improvement and deployment to plant apoplasmic and symplasmic compartments in plant tissues may help improve our ability to monitor metabolite levels after manipulating partitioning systems.

Although advances in our understanding of C and N partitioning systems allow us to test hypotheses by manipulating transporter streams from the “push,” “pull” and combined sides, it is also clear that our current understanding is limited when looking at the bigger picture of source/sink relationships. Moreover, complex metabolic interactions between assimilation and utilization of essential nutrients confounds attempts to engineer C and N partitioning. A systems biology approach that compares whole plant nutrient partitioning in high yielding domesticated plants and less productive ancestral varieties may shed light on the labyrinth of interconnected networks that were modified during crop domestication and improvement. Nested association mapping populations, genome wide association studies, and whole-genome re-sequencing are some of the tools that could identify genetic changes in key genes and quantitative trait loci (Meyer and Purugganan, 2013; Olsen and Wendel, 2013; Wallace et al., 2014). In addition, coordinated “omic” approaches could identify key changes in transcripts, proteins, and metabolites. In spite of the many advances in our understanding of the pathways of assimilate partitioning, we still lack fundamental insights into the control and regulation of this complex, system wide, physiological process in plants. Notwithstanding, the potential of developing targeted biomass strategies to optimize partitioning for practical yield gains in crops is an applied goal that supports and justifies continued basic science research in the fundamentals of C and N allocation.

### Conflict of Interest Statement

The authors declare that the research was conducted in the absence of any commercial or financial relationships that could be construed as a potential conflict of interest.
